# Supplementary material for: Pharmacogenetic strategies to mitigate cisplatin-induced ototoxicity in head and neck cancer: A cost-minimization analysis with the use of GSTP1 c.313A>G genotyping
Source: PLoS One. 2026 Apr 20;21(4):e0345371. doi: 10.1371/journal.pone.0345371 (PMC13095004; doi:10.1371/journal.pone.0345371)
Supplement: S1 File — (PDF) [file pone.0345371.s001.pdf]

Calculation of probabilities and costs based on the decision analytic model and transitional Markov states

Based on Bayes' theorem, allowing the incorporation of prior distributions for probability estimation in a specific scenario, the first-year follow-up costs can be calculated as follows, taking into account the decision analytic tree from **Figure 1**:

In the conventional treatment arm:

- (N * P2 * 0), for patients with loss to follow-up or death during treatment.
- N * P4 * (CDDP treatment price + investment in hearing aids), for patients with moderate or severe ototoxicity.
- N * (1 - P2 - P4) * CDDP treatment price, for asymptomatic patients.

Where N corresponds to the number of patients, and P is the probabilities to be calculated as stipulated in the analytical decision diagram above. The sum of these values corresponds to the estimated cost in the conventional arm.

In the intervention arm (genotype selection):

- N * (1 - P1) * P2 * genotyping price, for low-risk genotype group patients with loss to follow-up or death before treatment completion.
- N * (1 - P1) * P5 * (genotyping price + CDDP treatment price + investment in hearing aids), for low-risk genotype group patients with moderate to severe ototoxicity.
- N * (1 - P1) * (1 - P2 - P5) * (genotyping price + CDDP treatment price), for low-risk genotype group asymptomatic patients.
- N * P1 * P3 * genotyping price, for high-risk genotype group patients with loss to follow-up or death before treatment completion.
- N * P1 * P6 * (genotyping price + docetaxel treatment price + investment in hearing aids), for high-risk genotype group patients with moderate to severe ototoxicity.
- N * P1 * (1 – P3 – P6) * (genotyping price + CDDP treatment price), for high-risk genotype group asymptomatic patients.

Where N corresponds to the number of patients, and P is the probabilities to be calculated as stipulated in the analytical decision diagram. The sum of these values corresponds to the estimated cost in the intervention arm. For the difference between costs in the first year, the subtraction between the estimated costs of the conventional and intervention arms were performed.
