# Supplementary material for: Pharmacogenetic strategies to mitigate cisplatin-induced ototoxicity in head and neck cancer: A cost-minimization analysis with the use of GSTP1 c.313A>G genotyping
Source: PLoS One. 2026 Apr 20;21(4):e0345371. doi: 10.1371/journal.pone.0345371 (PMC13095004; doi:10.1371/journal.pone.0345371)
Supplement: S1 Table — (PDF) [file pone.0345371.s002.pdf]

Table S1. DNA Extraction Costs (in United States Dollars)

Reagent	Average Gross Cost	Quantity Used	Cost per Sample	10% Loss
Hemolysis Buffer 1	$0.68/1 L	24 mL	$0.02	$0.02
Hemolysis Buffer 2	$1.33/200 mL	1 mL	$0.01	$0.01
Digestion Buffer	$0.04/20 mL	400 µL	$0.00	$0.00
Proteinase K	$36.16/5 mL	20 µL	$0.14	$0.16
Lithium Chloride	$21.62/100 mL	200 µL	$1.08	$1.20
Absolute Ethanol	$27.02/1 L	1 mL	$0.03	$0.03
70% Ethanol	$18.91/1 L	2 mL	$0.04	$0.04
TE Buffer	$0.02/1 mL	30 µL	$0.00	$0.00
**Total**			**$1.32**	**$1.46**
