# Supplementary material for: Pharmacogenetic strategies to mitigate cisplatin-induced ototoxicity in head and neck cancer: A cost-minimization analysis with the use of GSTP1 c.313A>G genotyping
Source: PLoS One. 2026 Apr 20;21(4):e0345371. doi: 10.1371/journal.pone.0345371 (PMC13095004; doi:10.1371/journal.pone.0345371)
Supplement: S2 Table — (PDF) [file pone.0345371.s003.pdf]

Table S2. Real-Time PCR Costs (in United States Dollars)

Reagent	Average Gross Cost	Quantity Used	Total
Master Mix	$629.86/10 mL	$0.71 (10 µL)	$2.84 (40 µL)
Diluted Probe (Taqman®)	$335.61/376 µL	$1.00 (1 µL)	$4.00 (4 µL)
MicroAmp™ Tube (0.1 mL)	$127.01/125 units	$0.14 (1 tube)	$0.56 (4)
MicroAmp™ Optical Cap Strips	$158.93/300 units	$0.07 (1 cap)	$0.29 (4)
**Total**			**$9.65**
