# Supplementary material for: Pharmacogenetic strategies to mitigate cisplatin-induced ototoxicity in head and neck cancer: A cost-minimization analysis with the use of GSTP1 c.313A>G genotyping
Source: PLoS One. 2026 Apr 20;21(4):e0345371. doi: 10.1371/journal.pone.0345371 (PMC13095004; doi:10.1371/journal.pone.0345371)
Supplement: S3 Table — (PDF) [file pone.0345371.s004.pdf]

Table S3. Equipment involved in real-time PCR (in United States Dollars)

Equipment	Value
**StepOne Plus PCR System**	$38,340.00
**Amortization (20%)**	$7,668.00
