# Supplementary material for: Pharmacogenetic strategies to mitigate cisplatin-induced ototoxicity in head and neck cancer: A cost-minimization analysis with the use of GSTP1 c.313A>G genotyping
Source: PLoS One. 2026 Apr 20;21(4):e0345371. doi: 10.1371/journal.pone.0345371 (PMC13095004; doi:10.1371/journal.pone.0345371)
Supplement: S4 Table — (PDF) [file pone.0345371.s005.pdf]

Table S4. Manpower costs (in United States Dollars)

Labor Costs	Monthly Gross Salary
**Gross Salary**	$809.78
**Meal Voucher**	$239.21
**13th Month Salary Provision**	$67.48
**Vacation Provision**	$67.48
**Vacation Bonus Provision**	$22.49
**Severance Guarantee Fund**	$64.78
**Severance Guarantee Fund for Bonuses**	$12.60
**National Institute of Social Security (20% employer contribution)**	$161.96
**National Institute of Social Security for Bonuses**	$31.49
**Monthly Total (160 hours)**	**$1,477.27**
**Hourly Cost**	**$9.23**
**Cost per Minute**	**$0.15**
