# Supplementary material for: Pharmacogenetic strategies to mitigate cisplatin-induced ototoxicity in head and neck cancer: A cost-minimization analysis with the use of GSTP1 c.313A>G genotyping
Source: PLoS One. 2026 Apr 20;21(4):e0345371. doi: 10.1371/journal.pone.0345371 (PMC13095004; doi:10.1371/journal.pone.0345371)
Supplement: S5 Table — (PDF) [file pone.0345371.s006.pdf]

Table S5. Costs per PCR test, according to the number of samples evaluated (in United States Dollars)

Simultaneous Tests	1	2	3	4	5
**DNA Extraction (3.93 hours)**					
**Manpower**	$36.32	$18.16	$12.11	$9.08	$7.26
**Reagents**	$1.46	$1.46	$1.46	$1.46	$1.46
**Real-Time PCR (2.33 hours)**					
**Manpower**	$21.54	$10.77	$7.18	$5.39	$4.31
**Material**	$1.07	$0.64	$0.50	$0.43	$0.39
**Reagents**	$8.56	$5.13	$3.99	$3.42	$3.08
**Data Reporting (0.33 hours)**					
**Manpower**	$3.08	$1.54	$1.03	$0.77	$0.62
**Total**	$72.03	$37.70	$26.27	$20.55	$17.12
