# Supplementary material for: Pharmacogenetic strategies to mitigate cisplatin-induced ototoxicity in head and neck cancer: A cost-minimization analysis with the use of GSTP1 c.313A>G genotyping
Source: PLoS One. 2026 Apr 20;21(4):e0345371. doi: 10.1371/journal.pone.0345371 (PMC13095004; doi:10.1371/journal.pone.0345371)
Supplement: S6 Table — (PDF) [file pone.0345371.s007.pdf]

Table S6. Costs related to treatment (in United States Dollars)

Material/Service	Average Gross Cost	Quantity Used	Cost per Patient
**Cisplatin 100mg/m² every 21 days (3 cycles)**			
**Cisplatin 1mg/ml, 50 ml**	\$34.53	428 mg	\$147.78
**Dexamethasone 10 mg/2.5 ml**	\$3.36	30 mg	\$10.09
**Ondansetron 8 mg/4 ml**	\$1.45	24 mg	\$4.35
**Fosaprepitant 150 mg**	\$38.41	450 mg	\$115.23
**Mannitol 200 ml**	\$2.99	600 ml	\$8.98
**Equipment + Puncture Material**	\$1.42	3 sets + catheter	\$4.25
**0.9% Saline Solution 1000 ml**	\$2.72	6000 ml	\$16.33
**Total**			**\$307.02**
**Docetaxel 15mg/m² weekly (7 cycles)**			
**Docetaxel 80 mg/4 ml**	\$70.31	180 mg	\$158.19
**Dexamethasone 10 mg/2.5 ml**	\$3.36	70 mg	\$23.55
**Ondansetron 8 mg/4 ml**	\$1.45	56 mg	\$10.16
**Diphenhydramine 50 mg**	\$7.92	350 mg	\$55.44
**Equipment + Puncture Material**	\$1.42	7 sets + catheter	\$9.93
**0.9% Saline Solution 500 ml**	\$1.86	3500 ml	\$13.03
**Total**			**\$270.29**
