# Supplementary material for: Pharmacogenetic strategies to mitigate cisplatin-induced ototoxicity in head and neck cancer: A cost-minimization analysis with the use of GSTP1 c.313A>G genotyping
Source: PLoS One. 2026 Apr 20;21(4):e0345371. doi: 10.1371/journal.pone.0345371 (PMC13095004; doi:10.1371/journal.pone.0345371)
Supplement: S7 Table — (PDF) [file pone.0345371.s008.pdf]

**Table S7. Costs related to the use of hearing aids in patients with moderate to severe ototoxicity (in United States Dollars)**

| Material/Service | Average Cost | Annual Frequency | Annual Cost per Patient (1 Device) |
|---|---|---|---|
| **Amplifier (Type A)** | $190.91 | 1 every 3 years (unilateral or bilateral) | $63.64 |
| **Pure-tone audiometry** | $2.05 | 1 | $2.05 |
| **Medical consultation** | $4.50 | 1 | $4.50 |
| **Audiological evaluation** | $1.81 | 2 | $3.63 |
| **Total** | | | **$73.81** |
