# Supplementary material for: Pharmacogenetic strategies to mitigate cisplatin-induced ototoxicity in head and neck cancer: A cost-minimization analysis with the use of GSTP1 c.313A>G genotyping
Source: PLoS One. 2026 Apr 20;21(4):e0345371. doi: 10.1371/journal.pone.0345371 (PMC13095004; doi:10.1371/journal.pone.0345371)
Supplement: S8 Table — (PDF) [file pone.0345371.s009.pdf]

**Table S8. Cost savings between the conventional treatment group and genotyping, considering the number of simultaneous tests and the volume of patients analyzed**

| Patients | 1 Sample | 2 Samples | 3 Samples | 4 Samples | 5 Samples |
|---|---|---|---|---|---|
| **0** | -US$ 7,668.00 | -US$ 7,668.00 | -US$ 7,668.00 | -US$ 7,668.00 | -US$ 7,668.00 |
| **25** | -US$ 8,078.48 | -US$ 7,220.23 | -US$ 6,934.31 | -US$ 6,791.35 | -US$ 6,705.67 |
| **50** | -US$ 8,488.96 | -US$ 6,772.45 | -US$ 6,200.61 | -US$ 5,914.70 | -US$ 5,743.35 |
| **75** | -US$ 8,899.44 | -US$ 6,324.68 | -US$ 5,466.92 | -US$ 5,038.04 | -US$ 4,781.02 |
| **100** | -US$ 9,309.92 | -US$ 5,876.90 | -US$ 4,733.23 | -US$ 4,161.39 | -US$ 3,818.69 |
| **125** | -US$ 9,720.40 | -US$ 5,429.13 | -US$ 3,999.54 | -US$ 3,284.74 | -US$ 2,856.36 |
| **150** | -US$ 10,130.88 | -US$ 4,981.35 | -US$ 3,265.84 | -US$ 2,408.09 | -US$ 1,894.04 |
| **175** | -US$ 10,541.36 | -US$ 4,533.58 | -US$ 2,532.15 | -US$ 1,531.44 | -US$ 931.71 |
| **200** | -US$ 10,951.84 | -US$ 4,085.80 | -US$ 1,798.46 | -US$ 654.79 | US$ 30.62 |
| **225** | -US$ 11,362.32 | -US$ 3,638.03 | -US$ 1,064.76 | US$ 221.87 | US$ 992.95 |
| **250** | -US$ 11,772.80 | -US$ 3,190.25 | -US$ 331.07 | US$ 1,098.52 | US$ 1,955.27 |
| **275** | -US$ 12,183.28 | -US$ 2,742.48 | US$ 402.62 | US$ 1,975.17 | US$ 2,917.60 |
| **300** | -US$ 12,593.76 | -US$ 2,294.70 | US$ 1,136.31 | US$ 2,851.82 | US$ 3,879.93 |
| **325** | -US$ 13,004.23 | -US$ 1,846.93 | US$ 1,870.01 | US$ 3,728.47 | US$ 4,842.25 |
| **350** | -US$ 13,414.71 | -US$ 1,399.15 | US$ 2,603.70 | US$ 4,605.13 | US$ 5,804.58 |
| **375** | -US$ 13,825.19 | -US$ 951.38 | US$ 3,337.39 | US$ 5,481.78 | US$ 6,766.91 |
| **400** | -US$ 14,235.67 | -US$ 503.60 | US$ 4,071.08 | US$ 6,358.43 | US$ 7,729.24 |
| **425** | -US$ 14,646.15 | -US$ 55.83 | US$ 4,804.78 | US$ 7,235.08 | US$ 8,691.56 |
| **450** | -US$ 15,056.63 | US$ 391.94 | US$ 5,538.47 | US$ 8,111.73 | US$ 9,653.89 |
| **475** | -US$ 15,467.11 | US$ 839.72 | US$ 6,272.16 | US$ 8,988.39 | US$ 10,616.22 |
| **500** | -US$ 15,877.59 | US$ 1,287.49 | US$ 7,005.86 | US$ 9,865.04 | US$ 11,578.55 |

US$: United States Dollars
