# Supplementary material for: Pharmacogenetic strategies to mitigate cisplatin-induced ototoxicity in head and neck cancer: A cost-minimization analysis with the use of GSTP1 c.313A>G genotyping
Source: PLoS One. 2026 Apr 20;21(4):e0345371. doi: 10.1371/journal.pone.0345371 (PMC13095004; doi:10.1371/journal.pone.0345371)
Supplement: S9 Table — (PDF) [file pone.0345371.s010.pdf]

Table S9. Total costs per patient in the first year for the *GSTP1* c.313A>G genotyping group and the differences compared to the conventional arm, considering simultaneous genotyping of five samples

Patients	Cost (Median)	Credibility Interval	Difference (Median)	Credibility Interval
**25**	US$636.42	US$625.71 to US$645.97	-US$268.23	-US$274.92 to -US$266.52
**50**	US$483.06	US$472.35 to US$492.61	-US$114.87	-US$121.56 to -US$113.16
**75**	US$431.94	US$421.23 to US$441.49	-US$63.75	-US$70.44 to -US$62.04
**100**	US$406.38	US$395.67 to US$415.93	-US$38.19	-US$44.88 to -US$36.48
**125**	US$391.04	US$380.34 to US$400.59	-US$22.85	-US$29.55 to -US$21.14
**150**	US$380.82	US$370.11 to US$390.37	-US$12.63	-US$19.32 to -US$10.92
**175**	US$373.51	US$362.81 to US$383.07	-US$5.32	-US$12.02 to -US$3.62
**200**	US$368.04	US$357.33 to US$377.59	US$0.15	-US$6.54 to US$1.86
**225**	US$363.78	US$353.07 to US$373.33	US$4.41	-US$2.28 to US$6.12
**250**	US$360.37	US$349.67 to US$369.92	US$7.82	US$1.12 to US$9.53
**275**	US$357.58	US$346.88 to US$367.13	US$10.61	US$3.91 to US$12.32
**300**	US$355.26	US$344.55 to US$364.81	US$12.93	US$6.24 to US$14.64
**325**	US$353.29	US$342.59 to US$362.84	US$14.90	US$8.20 to US$16.61
**350**	US$351.61	US$340.90 to US$361.16	US$16.58	US$9.89 to US$18.29
**375**	US$350.15	US$339.44 to US$359.70	US$18.05	US$11.35 to US$19.75
**400**	US$348.87	US$338.16 to US$358.42	US$19.32	US$12.63 to US$21.03
**425**	US$347.74	US$337.04 to US$357.29	US$20.45	US$13.75 to US$22.16
**450**	US$346.74	US$336.03 to US$356.29	US$21.45	US$14.76 to US$23.16
**475**	US$345.84	US$335.14 to US$355.39	US$22.35	US$15.65 to US$24.06
**500**	US$345.03	US$334.33 to US$354.59	US$23.16	US$16.46 to US$24.87

US$: United States Dollars
