# Supplementary material for: Pharmacogenetic strategies to mitigate cisplatin-induced ototoxicity in head and neck cancer: A cost-minimization analysis with the use of GSTP1 c.313A>G genotyping
Source: PLoS One. 2026 Apr 20;21(4):e0345371. doi: 10.1371/journal.pone.0345371 (PMC13095004; doi:10.1371/journal.pone.0345371)
Supplement: S11 Table — (PDF) [file pone.0345371.s012.pdf]

**Table S11. Inferior limit of credibility results of *GSTP1* c.313A>G genotyping costs compared to conventional treatment over a ten-year period for a population of 250 patients (in United States Dollars)**

| Year | 1 | 2 | 3 | 4 | 5 | 6 | 7 | 8 | 9 | 10 | Total |
|---|---|---|---|---|---|---|---|---|---|---|---|
| Patients | 231 | 213 | 197 | 181 | 167 | 155 | 143 | 132 | 122 | 112 | |
| Device Cost (US$) | $59.59 | $55.80 | $52.25 | $48.93 | $45.82 | $42.91 | $40.18 | $37.62 | $35.23 | $32.99 | |
| Service Cost (US$) | $9.53 | $8.92 | $8.35 | $7.82 | $7.33 | $6.86 | $6.42 | $6.02 | $5.63 | $5.27 | |
| Unamortized Hearing Aids (Conventional Group) | 0.7 | 1.3 | 58.8 | 54.8 | 51.0 | 47.6 | 44.3 | 41.3 | 38.5 | 35.8 | |
| Appointments (Conventional Group) | 68 | 63 | 59 | 55 | 51 | 48 | 44 | 41 | 38 | 36 | |
| Unamortized Hearing Aids (Genotyping Group) | 0.9 | 1.7 | 15.1 | 14.7 | 14.2 | 13.7 | 13.2 | 12.7 | 12.1 | 11.6 | |
| Appointments (Genotyping Group) | 16 | 16 | 15 | 15 | 14 | 14 | 13 | 13 | 12 | 12 | |
| Difference in Hearing Aids | -0.2 | -0.4 | 43.6 | 40.1 | 36.9 | 33.9 | 31.1 | 28.6 | 26.3 | 24.2 | |
| Difference in Appointments | 52 | 47 | 44 | 40 | 37 | 34 | 31 | 29 | 26 | 24 | |

| Year | 1 | 2 | 3 | 4 | 5 | 6 | 7 | 8 | 9 | 10 | Total |
|---|---|---|---|---|---|---|---|---|---|---|---|
| Cost Reduction in Hearing Aids (US$) | $-12.98 | $-22.39 | $2,280.19 | $1,962.52 | $1,689.11 | $1,453.78 | $1,251.25 | $1,076.93 | $926.89 | $797.76 | $11,403.04 |
| Cost Reduction in Appointments (US$) | $492.15 | $423.58 | $364.57 | $313.78 | $270.06 | $232.44 | $200.06 | $172.19 | $148.20 | $127.55 | $2,744.57 |
| Total Reduction (US$) | $479.16 | $401.19 | $2,644.76 | $2,276.30 | $1,959.17 | $1,686.22 | $1,451.30 | $1,249.11 | $1,075.09 | $925.31 | $14,147.61 |
| Average Reduction per Patient (US$) | $2.08 | $1.88 | $13.45 | $12.55 | $11.70 | $10.91 | $10.17 | $9.49 | $8.84 | $8.25 | |

US$: United States Dollars
