# Supplementary material for: Pharmacogenetic strategies to mitigate cisplatin-induced ototoxicity in head and neck cancer: A cost-minimization analysis with the use of GSTP1 c.313A>G genotyping
Source: PLoS One. 2026 Apr 20;21(4):e0345371. doi: 10.1371/journal.pone.0345371 (PMC13095004; doi:10.1371/journal.pone.0345371)
Supplement: S12 Table — (PDF) [file pone.0345371.s013.pdf]

Table S12. Superior limit of credibility results of *GSTP1* c.313A>G genotyping costs compared to conventional treatment over a ten-year period for a population of 250 patients (in United States Dollars)

Year	1	2	3	4	5	6	7	8	9	10	Total
Patients	231	213	197	181	167	155	143	132	122	112	
Device Cost (US$)	$59.59	$55.80	$52.25	$48.93	$45.82	$42.91	$40.18	$37.62	$35.23	$32.99	
Service Cost (US$)	$9.53	$8.92	$8.35	$7.82	$7.33	$6.86	$6.42	$6.02	$5.63	$5.27	
Unamortized Hearing Aids (Conventional Group)	0.6	1.1	82.1	76.2	70.7	65.7	60.9	56.6	52.5	48.7	
Appointments (Conventional Group)	95	88	82	76	71	66	61	57	53	49	
Unamortized Hearing Aids (Genotyping Group)	0.7	1.4	48.0	44.9	42.0	39.2	36.7	34.3	32.0	29.9	
Appointments (Genotyping Group)	55	51	48	45	42	39	37	34	32	30	
Difference in Hearing Aids	-0.2	-0.3	34.0	31.3	28.7	26.4	24.3	22.3	20.5	18.8	
Difference in Appointments	40	37	34	31	29	26	24	22	21	19	

Year	1	2	3	4	5	6	7	8	9	10	Total
Cost Reduction in Hearing Aids (US$)	$-10.12	$-17.45	$1,777.09	$1,529.51	$1,316.42	$1,133.02	$975.17	$839.31	$722.38	$621.74	$8,887.07
Cost Reduction in Appointments (US$)	$383.56	$330.12	$284.13	$244.55	$210.48	$181.15	$155.92	$134.19	$115.50	$99.41	$2,139.01
Total Reduction (US$)	$373.44	$312.67	$2,061.22	$1,774.05	$1,526.90	$1,314.17	$1,131.09	$973.51	$837.88	$721.15	$11,026.07
Average Reduction per Patient (US$)	$1.62	$1.47	$10.49	$9.78	$9.12	$8.50	$7.93	$7.39	$6.89	$6.43	

US$: United States Dollars
