# Supplementary material for: Pharmacogenetic strategies to mitigate cisplatin-induced ototoxicity in head and neck cancer: A cost-minimization analysis with the use of GSTP1 c.313A>G genotyping
Source: PLoS One. 2026 Apr 20;21(4):e0345371. doi: 10.1371/journal.pone.0345371 (PMC13095004; doi:10.1371/journal.pone.0345371)
Supplement: Attachment — Submitted filename: reviewer_letter.docx [file pone.0345371.s014.docx]

**Ref.: Manuscript ID PONE-D-25-07423**

Dear Editors and Reviewers,

We sincerely thank you for your thoughtful and constructive feedback. We have carefully revised the manuscript in response to the reviewers' comments, and we believe that the changes have improved the clarity, robustness, and transparency of our analysis. Below, we provide a detailed point-by-point response. All changes are highlighted in yellow in the revised manuscript.

- Reviewer 1:

“This paper has been written after a thorough analysis after implementation of statistical tools and and a deep analysis of cost minimization has been provided. The author have examined all the areas that needed to be addressed.”

Dear reviewer, we sincerely thank you for your thoughtful comment.

- Reviewer 2:

Dear reviewer, we appreciate your suggestions, which certainly helped enhance the clarity of this study . Please find below a detailed description of the revisions made in response to your comments. All changes have also been highlighted in the revised manuscript.

“Clarify the assumption of oncologic equivalence between cisplatin and docetaxel and how it may influence cost conclusions.”

We have included further descriptions on the available evidence of efficacy for docetaxel as a radiosensitizer in the *Introduction* section, page 4, lines 74-80, as well as *Discussion*, page 21, lines 394-396. Even though there are no direct head-to-head specific comparisons of cisplatin and docetaxel as single agent radiosensitizers in head and neck squamous cell carcinoma chemoradiation, there is sufficient evidence regarding the use of docetaxel as an alternative agent, and robust results in a randomized trial comparing cisplatin or docetaxel in combination with cetuximab. The assumption of equivalence as a limitation is addressed in the *Discussion*, page 21, line 384, as in the oroiginal version.

“Discuss the potential variability in patient adherence to hearing aid treatment and follow-up, which may impact cost estimations.”

Great suggestion! In this revised version, we added the variability of adherence rates as a limitation to the interpretation of the results (page 22, lines 417-421).

“Justify the use of pediatric data in adult modeling with more nuance or discuss its limitations more explicitly”

We would like to clarify that the data from the pediatric cohort were not applied as a prior in the model, due to potential differences inherent to that population. To avoid any misunderstanding caused by the previous wording, we have improved the description of the modelling choice in greater detail (page 14, lines 276–282).

“Consider a sentence or two on why QALYs were not modeled and what implications that may have for future research or broader decision-making.”

We added a justification in the *Discussion* for not including QALYs in this analysis (page 19, lines 344-349). We now acknowledge that including quality-adjusted life years could enhance generalizability and support broader health policy applications in future studies.

“Simplify abstract language and add brief clarifications of acronyms (CDDP, PCR) on first use.”

We revised the *Abstract* to enhance clarity and readability. Additionally, we reviewed all abbreviations and acronyms to ensure they are properly defined at first use.

We are grateful for the opportunity to improve the manuscript and thank the editorial team and reviewers for their time and guidance.

Yours sincerely,
